# Real-Time Ventricular Cancellation in Unipolar Atrial Fibrillation Electrograms

**DOI:** 10.3389/fbioe.2020.00789

**Published:** 2020-07-30

**Authors:** Gonzalo R. Ríos-Muñoz, Antonio Artés-Rodríguez, Francisco Fernández-Avilés, Ángel Arenal

**Affiliations:** ^1^Instituto de Investigación Sanitaria Gregorio Marañón (IiSGM), Madrid, Spain; ^2^La Red de Terapia Celular (TerCel), Instituto de Salud Carlos III, Madrid, Spain; ^3^Departamento de Cardiología, Hospital General Universitario Gregorio Marañón, Madrid, Spain; ^4^Departamento de Bioingeniería e Ingeniería Aeroespacial, Universidad Carlos III de Madrid, Madrid, Spain; ^5^Departamento de Teoría de la Señal y Comunicaciones, Universidad Carlos III de Madrid, Madrid, Spain; ^6^Center for Biomedical Research in Cardiovascular Disease Network (CIBERCV), Madrid, Spain; ^7^Facultad de Medicina, Universidad Complutense de Madrid, Madrid, Spain

**Keywords:** atrial fibrillation, unipolar electrograms, real-time, biomedical signal processing, multi-electrode catheter

## Abstract

Unipolar atrial fibrillation (AF) electrograms (EGMs) require far-field ventricle cancellation to recover hidden atrial activations. Current methods cannot achieve real-time cancellation because of the temporal delay they introduce. We propose a new real-time ventricular cancellation (RVC) method based on causal implementation optimized for real-time functioning. The method is similar to the classical average beat subtraction (ABS) method but it computes the ventricular contribution before the ventricular activation finishes. We compare the proposed method to the ABS on synthetic and real EGM databases for the time and frequency domains. All parameters and their optimal values are analyzed and validated. The RVC method provides a good reconstruction of the unipolar EGMs and a better local activation time detection than the classical approach with average F1scores 0.7307 and 0.7125, respectively. The spectral analysis shows that the average power after ventricular cancellation is reduced for frequency bands between 3 and 5.5 Hz, demonstrating that the proposed method removes the ventricular component present in the unipolar EGM signals compared to the ABS method. The phase mapping analysis on the RVC method presented lower error when comparing the annotated EGM cycles with the phase inversion intervals. In terms of performance ABS and RVC behave similarly, but the real-time capability of the latter justifies its preference over the offline implementations. In the clinical environment other online investigations, e.g., rotational activity assessment, dominant frequency or local activation time mapping, might benefit from the real-time potential of the proposed cancellation method.

## 1. Introduction

Atrial fibrillation (AF) is a cardiac arrhythmia characterized by irregular activations of the upper chambers of the heart, the atria. The disorganized electrical activity prevents the atria to deliver blood to the ventricles efficiently. Although AF is the most common arrhythmia in the clinical practice treatments remain suboptimal, in part due to the poor understanding of its initiating and perpetuating mechanisms (Kirchhof et al., 2016). To study AF electrophysiologists employ multi-electrode catheters advanced inside the atrial chambers. The catheters contain multiple electrodes that register voltage electrograms (EGMs) in contact with the atrial tissue, providing a measurement of the electrical activity of AF.

Signal processing algorithms applied on the EGMs provide useful insight into the mechanistical properties of AF, but the irregularity of the AF activation patterns in time makes it difficult to analyze their behavior (Ciaccio et al., 2011; Kogawa et al., 2015). To study AF current methods rely on local activation times (LATs) in unipolar EGMs to address the detection of anomalous propagation patterns, i.e., rotors or focal electrical discharges (Narayan et al., 2012; Daoud et al., 2017). In unipolar EGMs each activation is defined by a sharp deflection of voltage, and LATs can be identified at the maximum negative derivative of the signal $-\frac{dV}{dt}$, independently of the direction of the propagating wavefront (Spach et al., 1979). However, unipolar EGMs also record far-field ventricular signal, which interferes with the atrial activity.

To overcome this problem the classical approach is to apply the average beat subtraction (ABS) method (Slocum et al., 1985; Shkurovich et al., 1998), which considers the ventricular activity to be uncoupled to AF. The method detects the ventricular activity in a single electrocardiogram (ECG) lead, calculates an average beat template of the contribution of the ventricular activity, and subtracts it from the unipolar EGM revealing the hidden atrial activity. Variations to the ABS method have been proposed using different interpolation approaches for the subtracted QRST interval (Ahmad et al., 2011), or finer alignment matching different pattern lengths (Salinet et al., 2013; Ríos-Muñoz et al., 2018).

However, these methods require signal acquisitions from several seconds to minutes, e.g., 7, 30 s, or even 2 min (Narayan et al., 2012; Salinet et al., 2013; Daoud et al., 2017), and they are applied offline, with a chance for the AF to have changed at the time the results are available. Hence the need to process the signals in real-time and also to reduce the overall procedure time for safety reasons regarding the life of the patient.

To address this problem we present a new method that applies real-time ventricular cancellation (RVC) on short EGM segments, i.e., 100 ms, to achieve real-time signal processing, as an alternative to other methods that work with longer acquisitions. Our method relies on the duration and morphology of the QT ECG signal, which at rest remains stable (El-Chami et al., 2010; Vančura et al., 2016). Hence, we can store previous EGMs to estimate the ventricle pattern. We subtract the far-field contribution using this pattern in real-time even for the segments that only contain a portion of the total QT interval. We align the segment with the pattern and we subtract only the portion of the pattern related to the segment. We do not require a complete QT interval to apply our method, reducing the delay between acquisition and result availability, as opposed to Shkurovich et al. (1998), Ahmad et al. (2011), and Salinet et al. (2013). Additionally, the pattern adapts in time as new ventricular activations are detected evolving with changes in the fibrillatory state.

The real-time feature of the RVC method reduces the time to provide processed signals to other existing technologies and contributes to improve the understanding of the AF problem, in addition to other studies that also employed unipolar EGMs (Allessie et al., 2010; de Groot et al., 2010).

## 2. Materials and Methods

In this section we detail the methodology of the study. We first present the synthetic and real AF signal databases that we employed to analyze and validate the method. Second of all, we describe the RVC method and its implementation in the clinical practice. To that end, we estimated the storage requirements for the data buffers to achieve real-time signal processing and we analyzed the ventricular pattern quality. Finally, we introduce the time and frequency domain analysis to compare the ventricular cancellation performance of the RVC and ABS methods.

### 2.1. Atrial Fibrillation EGMs Databases

#### 2.1.1. Synthetic AF EGMs Database

**Figure 1:**
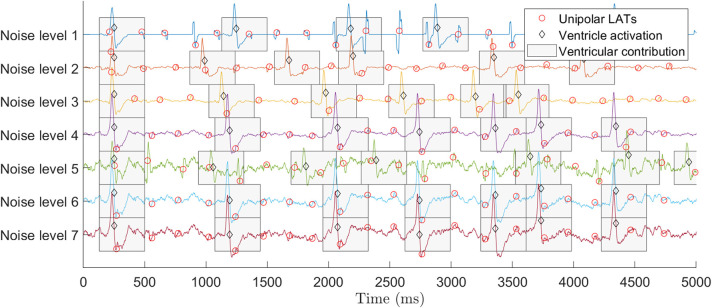
Synthetic EGM database signals for different levels of noise (Valinoti et al., 2018). Top signal corresponds to the presence of no noise (level 1) and bottom signal to the highest noise value (level 7). All signals include LATs and ventricular activations annotations. To improve the visualization only the first 5 s of the signals are displayed.

We employed a synthetic unipolar EGM database to analyze and set the values of the parameters of the RVC method (Valinoti et al., 2018). We also used the signals to compare the cancellation performance to the ABS method for LAT detection in the unipolar EGMs. The database consists of seven sets of 50 different unipolar atrial EGMs of 10 s length. The sets differ from each other by an additive noise signal that is generated by modifying the variance value of a Gaussian random variable, zero mean and variance σ_*n*_ = *j*·σ, where *n* ∈ [1, 7] noise levels, and *j* = 0, 0.1, 0.2, 0.5, 0.7, 0.9, 1. The value of σ is obtained by the autoregressive model in Valinoti et al. (2018). The different levels of noise are useful for testing and validating the performance and robustness of the algorithm at challenging situations, e.g., EGMs exhibiting low signal to noise ratio or fragmentation. The database includes the annotated LATs location for all the unipolar EGMs, and it contains ventricular far-field which is also delimited and known for all the signals. Figure 1 provides an example of the synthetic signals in the database for the different noise levels including LAT and ventricular far-field annotations.

#### 2.1.2. Real AF EGMs Database

The real AF signals in the study were acquired and processed on 32 AF patients. All patients gave full informed consent and the study was approved by the Institutional Review Board of the center. The sampling frequency was *f*_*s*_ = 1 KHz, unipolar EGMs referred to the Wilson central terminal were filtered 0.05–100 Hz, bipolar EGMs 30–240 Hz, and ECGs 0.05–100 Hz.

Each acquisition consists of one ECG signal and 15 unipolar EGMs recorded with a PentaRay catheter. A total of 890 acquisitions were made (mean 27.81 acquisitions per patient), i.e., 890 ECG and 13,350 unipolar EGM signals in the database. The acquisitions were recorded at different regions of the left atrium during the routine electro-anatomical mapping study (Carto 3 system, Biosense Webster) prior to catheter ablation of the pulmonary veins. The database includes signals from: pulmonary veins, left atrial appendage, roof wall, posterior wall, septum, ridge, mitral ring, and lateral wall. All signals were 10 s long at a sampling frequency *f*_*s*_ = 1 KHz.

### 2.2. Real-Time Ventricle Cancellation Proposed Method

**Figure 2:**
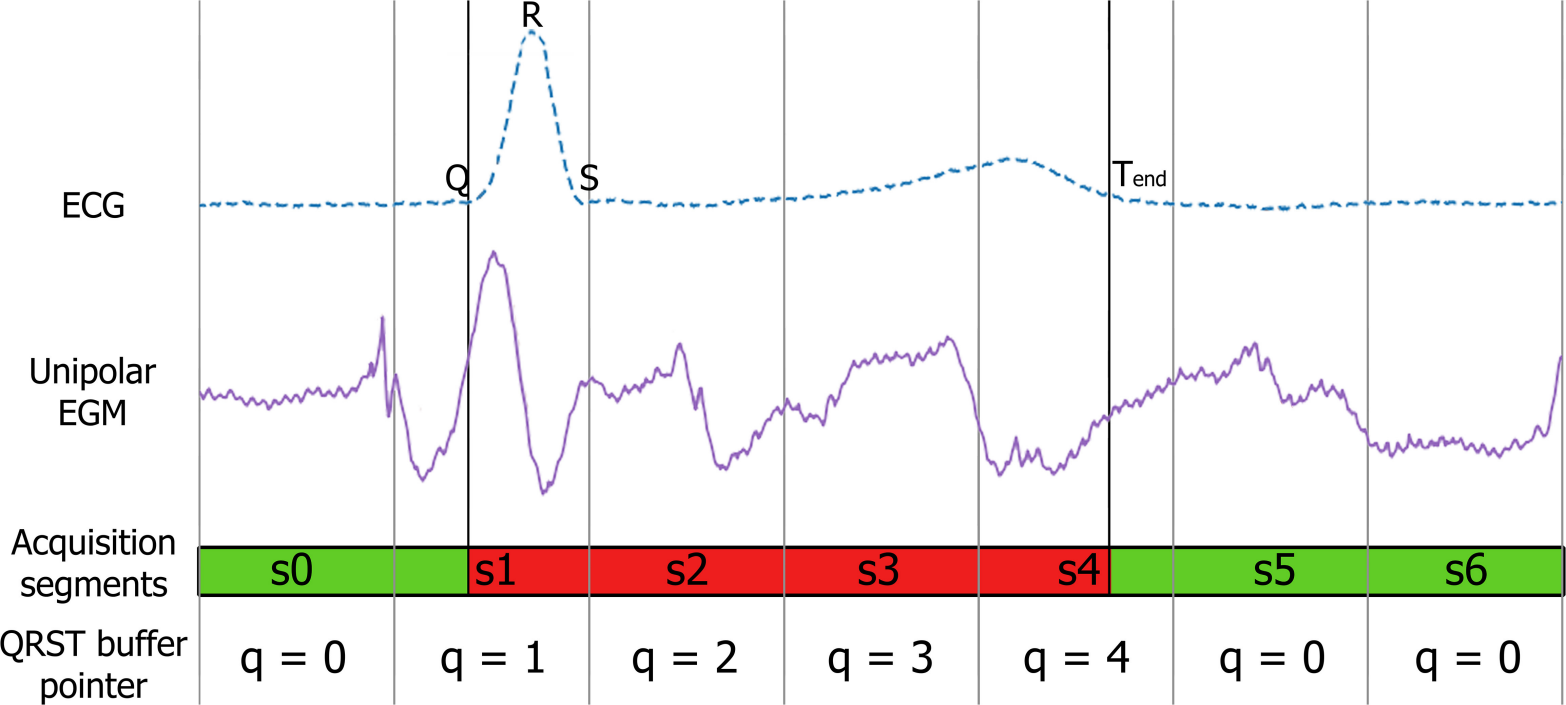
ECG and EGM containing far-field ventricular contribution. From top to bottom: reference ECG to narrow the ventricular component, unipolar EGM affected by the far-field ventricular activity, and 100 ms acquisitions illustrating the segments affected (red) and not affected (green) by the ventricular contribution. Fiducial points corresponding to the Q-peak, R-peak and *T*_*end*_ time instants are annotated in the ECG signal.

Ventricular cancellation methods require complete ventricular contributions to be applied. Online signal processing deals with smaller signal segments, e.g., 100 ms segments, hence no complete QRST complex is guaranteed in an acquisition, as Figure 2 shows. The ECG and EGM signals consist of seven consecutive segments, i.e., *s*_0_ − *s*_6_. In the figure, segments *s*0 and *s*5 − *s*6 are free of ventricular activity (in green), while *s*1−*s*4 are partially or fully affected (in red). Current cancellation methods narrow the temporal window affected by the ventricular contribution by knowing when the T-wave finishes (*T*_*end*_), and they remove the ventricle signal, that spans until segment *s*4 in Figure 2. To overcome this situation, we propose a RVC method that estimates the ventricular pattern contribution based on previous activations, and automatically updates the pattern when a new complete ventricular activation is available. The RVC can therefore be applied before *T*_*end*_ is acquired. Notice that in Figure 2 the R-peak appears in segment *s*1, 3 acquisitions before *T*_*end*_ finishes in *s*4.

The predictive nature of the method relies on R-peak detections in the ECG signal to trigger the online cancellation. The R-peak is detected based on a maximum peak voltage search in the ECG segment and imposing two conditions. First condition requires a blanking period between R-peaks, i.e., 300 ms, to prevent any spurious artifact or the T-wave to be misidentified as an R-peak. The second condition applies a minimum threshold that directly depends on the last *V* ventricular activations, and it is recalculated after every new R-peak detection. This threshold is adjusted to meet the ECG morphology of the patient (Supplementary Figure 1). The Q-peak is directly detected as the minimum value in a 50 ms window before the R-peak. We considered other methods in the literature based on wavelet transform, and Gilbert transform for R-peak detection. However, the high signal-to-noise ratio that the electrophysiology amplifier offered when acquiring the signals, and the simplicity of the proposed R-peak detection justified or preference for the R-detection method.

The *T*_*end*_ time instant is calculated with a method designed for positive T-waves by Zhang et al. (2006). In a 12-lead ECG several leads provide a positive T-wave, e.g., II, V5, or V6. The ECG exhibiting a significant larger R-peak than the T-wave amplitude is selected prior to the initialization of the system, as other electrophysiology and electro-anatomical mapping systems do (Narayan et al., 2012; Daoud et al., 2017). Supplementary Figure 1 further illustrates the fiducial point detection for continuous acquisitions in real-time in a patient.

The position of the R-peak varies within the segments, and the previous acquisition can contain part of the QR interval. Hence the RVC method buffers the previous signal acquisitions. For each channel we buffer the unipolar EGM signal segments, *Q* × *L* samples, where *L* is the acquisition segment size and *Q* the number of segments of length *L* to be stored in the buffer. Then, we estimate the ventricle pattern as the average of the last *V* stored ventricle activations. We take advantage of the low variability between consecutive ventricular contributions to set the value of *Q* segments (El-Chami et al., 2010). *Q* can be adequately adapted to the electrophysiological QRST duration of the patient, e.g., *Q* = 5–6 segments.

**Figure 3:**
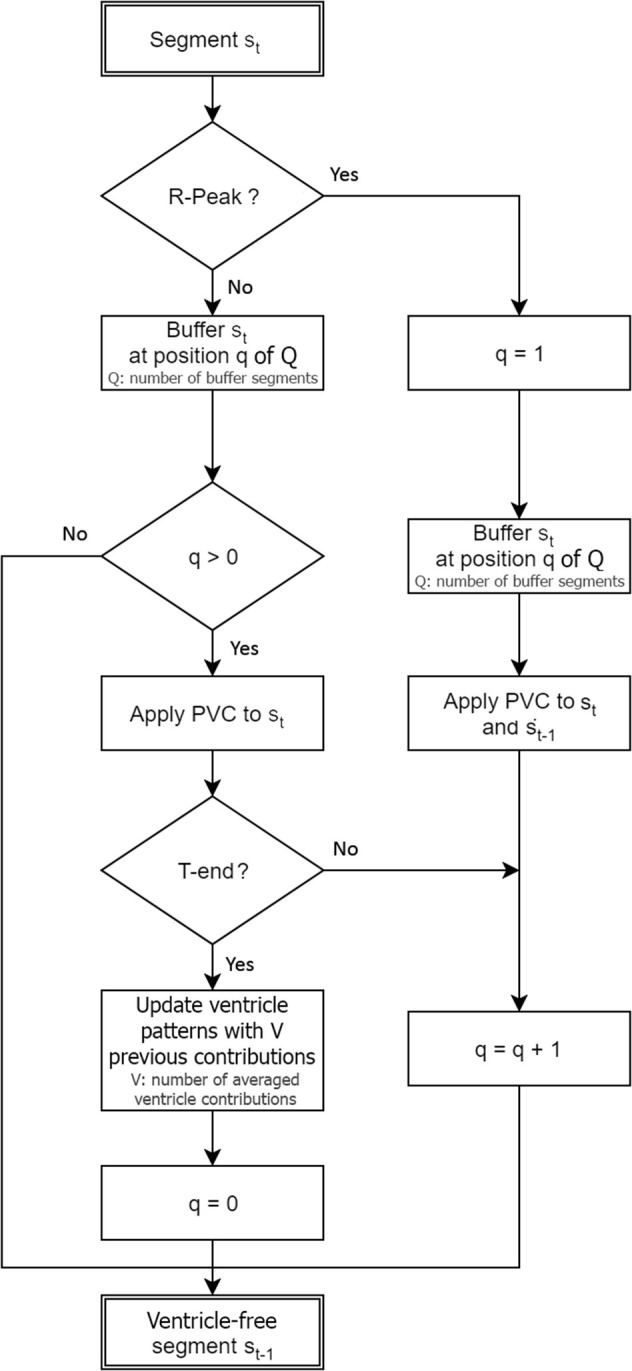
Real-time ventricle cancellation diagram flow for an EGM segment *s*_*t*_ acquired at time instant *t*.

The steps of the RVC method are simplified in the diagram flow in Figure 3. As a result of buffering the signals, at time *t* the method provides the cancellation for the signal *s*_*t*−1_ at *t* − 1, and the output for *s*_*t*_ is available at time instant *t* + 1, as Figure 3 depicts. We associate each segment *s*_*t*_ with a pointer to the QRST buffer where the signal containing the ventricular contribution is to be stored, namely *q* ∈ [0, *Q* − 1]. Following the diagram flow several cases occur whether a new R-peak is detected in the ECG segment or if the signal falls in the QRST range:

- Case *q* = 0 and no R-peak in the current segment: we store the EGM segment *s*_*t*_ in the QRST buffer at position *q* = 0 in the event the next segment contains one R-peak. Segment *s*_*t*−1_ is provided free of ventricle cancellation.
- Case *q* = 0 and R-peak present in the current segment: a new R-peak is detected, *q* is set to 1, and segment *s*_*t*_ is stored at position *q* = 1. We search for the Q-peak in the segments *s*_*t*−1_ and *s*_*t*_, and predictive ventricle cancellation is applied to segments *s*_*t*−1_ and *s*_*t*_. The pre-computed ventricle pattern is aligned by direct correlation with respect to the current signal in the QR interval and subtraction is performed. Segment *s*_*t*−1_ is provided at the output, and buffer storing pointer gets incremented, i.e., *q* = *q* + 1.
- Case 2 ≤ *q* ≤ *Q* − 1: similarly, segment *s*_*t*_ is stored at position *q* in the QRST buffer and ventricle cancellation is applied to it. We search for the *T*_*end*_ fiducial point and if not found *q* = *q* + 1. If found, the ventricular pattern is updated, we remove the oldest QRST contribution in the QRST buffer and compute the new pattern with the *V* − 1 remaining contributions plus the new contribution. Finally, the pointer is reinitialized to *q* = 0, ventricular free segment *s*_*t*−1_ is provided, and the RVC starts again, ready to detect the next R-peak.

### 2.3. Signal Buffering for Real-Time Achievement

**Figure 4:**
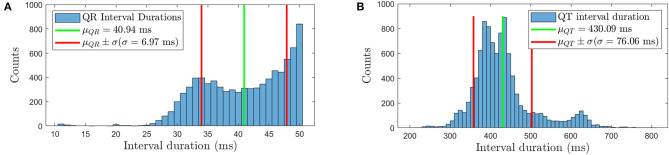
**(A)** QR duration histogram for the real AF ECG signals, **(B)** histogram of the duration of the QT intervals for the same signals. The mean plus/minus one standard deviation interval is also represented in both figures.

The RVC method requires to buffer the signals to apply the cancellation in real-time. To provide evidence that the Q-peak is contained in the proposed buffered signal, i.e., 100 ms, we studied the QR interval duration of all the database ECGs. Figure 4A shows the histogram for the QR duration of 9,836 ventricular activations in 890 ECGs from the real AF EGM database. The mean QR interval is 40.94 ms with a standard deviation of 6.97 ms. The QR interval range justifies the choice of 100 ms data segments, even for the most extreme case we encountered for a patient with a QR interval equals 50 ms.

Additionally, the QRST duration was analyzed to set the buffer size *Q* for the EGMs to be averaged. In the histogram in Figure 4B, the average interval lasts 430.09 ms with standard deviation equals to 76.06 ms, based on 9,836 detected QRST activations. Therefore a buffer of *Q* = 6 segments (600 ms) can store the unipolar EGM segments comprising the ventricular contribution. In the event of longer QRST intervals this buffer can be extended to match the needs of the patient.

### 2.4. Signal Conditioning and Implementation

Prior to apply the RVC method the signals are conditioned. A causal 500 ms median filter removes the respiratory movement of the patient present in unipolar EGMs as in de Chazal et al. (2003). Since the filter length is longer than the acquired segments, the signals are stored in a buffer so we can apply the median filter. The buffer size is *B* × *L* samples, where *L* is the acquisition segment size and *B* the number of segments of length *L*. For *L* = 100 ms acquisitions and a 500 ms median filter we set *B* = 5 segments. The filtering is performed sequentially for each channel. After the baseline is removed, the oldest 100 ms buffered segments are discarded and updated with the next acquired segments. Hence, the pre-processing step can be applied for filter orders greater than the acquisition segment length. The reader is referred to the frequency response function of the implemented median filter in Figure 4B in the article (Ríos-Muñoz et al., 2018) that further describes how the frequency components of the signals are affected.

The RVC method was implemented in Python 3.7, C, and employs the NVIDIA GPU Computing Toolkit v.10.0. The implementation is open source and no license dependent of any other commercial software. This grants feasibility and reproducibility for the software to be implemented in other systems at no supplementary license costs. A Graphical user interface (GUI) in python is in charge of calling the C and CUDA kernel functions for the real-time signal processing, and atrial signals free of ventricle contributions are available for display or further post-processing.

The hardware specifications of the system are: one 8-core Intel® Xeon® CPU 3.40 GHz processor, 16 GB RAM, on 64 bits Windows 7 Professional. The graphical processing unit (GPU) is one Nvidia Tesla K20c with 2496 CUDA cores, and a Titan V model was used for validating and testing the methods. The signals were acquired and digitalized using an analog to digital converter (ADC) manufactured by National Instruments connected to a ClearSign™ amplifier (Boston Scientific) which offers 16 analog output signals.

To achieve real-time results, we used short segments of unipolar EGM and ECG signals, with a duration of *L* = 100 ms which is the minimum segment size the ADC hardware offered (National Instruments, USA). Since the signal amplifier only offers 16 signals to the ADC, we selected one ECG lead and 15 unipolar electrode channels of the total 20 electrodes of the catheter to apply the RVC method.

We ran 500 continuous 100 ms segment acquisitions for 15 EGMs and one reference ECG using Python 3.7 and C dll functions. The averaged execution time for the code was 1.482 ms with standard deviation equals 0.546 ms. We emulated the acquisition and signal processing for an increasing number of virtual EGMs ranging from 15 to 200 channels. Supplementary Figure 2 shows the average execution time performance for different number of channels. The results provide evidence that the RVC method works in real-time since it processes the data faster than the 100 ms acquisition period, even for 200 simultaneous channels.

### 2.5. Estimated Ventricular Pattern Analysis

**Figure 5:**
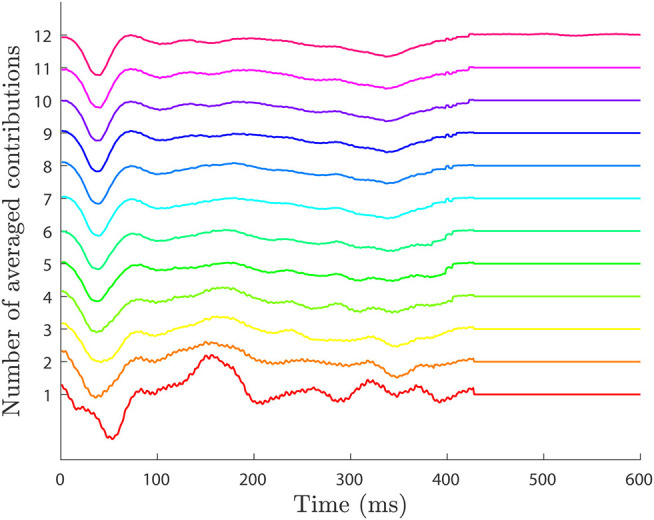
Ventricular pattern morphology for different number of averaged ventricular contributions. Patterns are obtained by averaging consecutive contributions ranging from *V* = 1 contribution (bottom) to *V* = 12 total contributions in the whole ECG signal (top). Higher number of contributions reveal the ventricular contribution, while for lower values the pattern contains stronger atrial component.

The stability of the pattern in time plays an important role in the RVC method. The number of ventricle contributions to provide a stable pattern is important regarding its implementation in the clinical practice. For each of the signals in both databases, incremental patterns were obtained by cumulatively averaging the number of contributions, namely *p*_*V*_, where *V* ∈ [1, *v*_*i*_], and *v*_*i*_ is the number of ventricle contributions in the *i* − *th* acquisition for *i* ∈ [1, 350] in the synthetic database, and *i* ∈ [1, 890] in the real AF EGM database. As an example, *p*_3_ is obtained as the average of three consecutive EGM segments affected by ventricle contribution. Figure 5 shows the averaged patterns for *V* = 1–12 contributions, the total number of QRST events in a real AF signal. The pattern for only one contribution contains strong atrial activity, see Figure 5 bottom signal. On the other hand, as the number of averaged contributions increases, the atrial component fades and the ventricular contribution is enhanced, which is the main foundation of ventricular cancellation methods.

To set the value of *V* that produces the most stable pattern, the quality of the patterns with respect to the number of contributions employed was measured. The mutual information for consecutive patterns was calculated, i.e., *I*(*p*_*j*_; *p*_*j*−1_) for *j* ∈ [2, *v*_*i*_] (Cover and Thomas, 2006). Mutual information measures how much information one signal tells us about another, namely *I*(*p*_*j*_; *p*_*j*−1_) = *H*(*p*_*j*_) − *H*(*p*_*j*_|*p*_*j*−1_). Particularly, how much new information a pattern with more ventricular contributions provides given one computed with less components. The function *H*(*p*_*j*_) is the entropy of pattern *p*_*j*_, and *H*(*p*_*j*_|*p*_*j*−1_) is the conditional entropy of pattern *p*_*j*_ given *p*_*j*−1_. If the signals are independent, i.e., very different, *H*(*p*_*j*_|*p*_*j*−1_) ≈ *H*(*p*_*j*_) hence *I*(*p*_*j*_; *p*_*j*−1_) ≈ 0. If they are similar, *H*(*p*_*j*_; *p*_*j*−1_) ≈ 0, and *I*(*p*_*j*_; *p*_*j*−1_) is maximized.

To reinforce the pattern analysis the Pearson's correlation coefficient measurement on the patterns is included. The correlation coefficient is calculated over two vectors, i.e., two patterns, for an increasing number of averaged ventricular contributions, similarly to the mutual information study. The correlation coefficient of two random variables measures their linear dependence, i.e., two patterns in our case *p*_*i*_ and *p*_*i*+1_. For each pattern of length equals *L* samples, the Pearson's correlation coefficient is defined as

(1) $$\begin{array}{l} r_{p_{i},p_{i+1}}=\frac{\sum_{n=1}^{L}\left(p_{i}\left[n\right]-\bar{p_{i}}\right)\left(p_{i+1}\left[n\right]-\bar{p_{i+1}}\right)}{\sqrt{\sum_{n=1}^{L}{\left(p_{i}\left[n\right]-\bar{p_{i}}\right)}^{2}}\sqrt{\sum_{n=1}^{L}{\left(p_{i+1}\left[n\right]-\bar{p_{i+1}}\right)}^{2}}}. \end{array}$$

where *n* refers to the time instant *n* ∈ [1, *L*] samples, and $\bar{p_{i}}$ and $\bar{p_{i+1}}$ are the mean values of the patterns.

**Figure 6:**
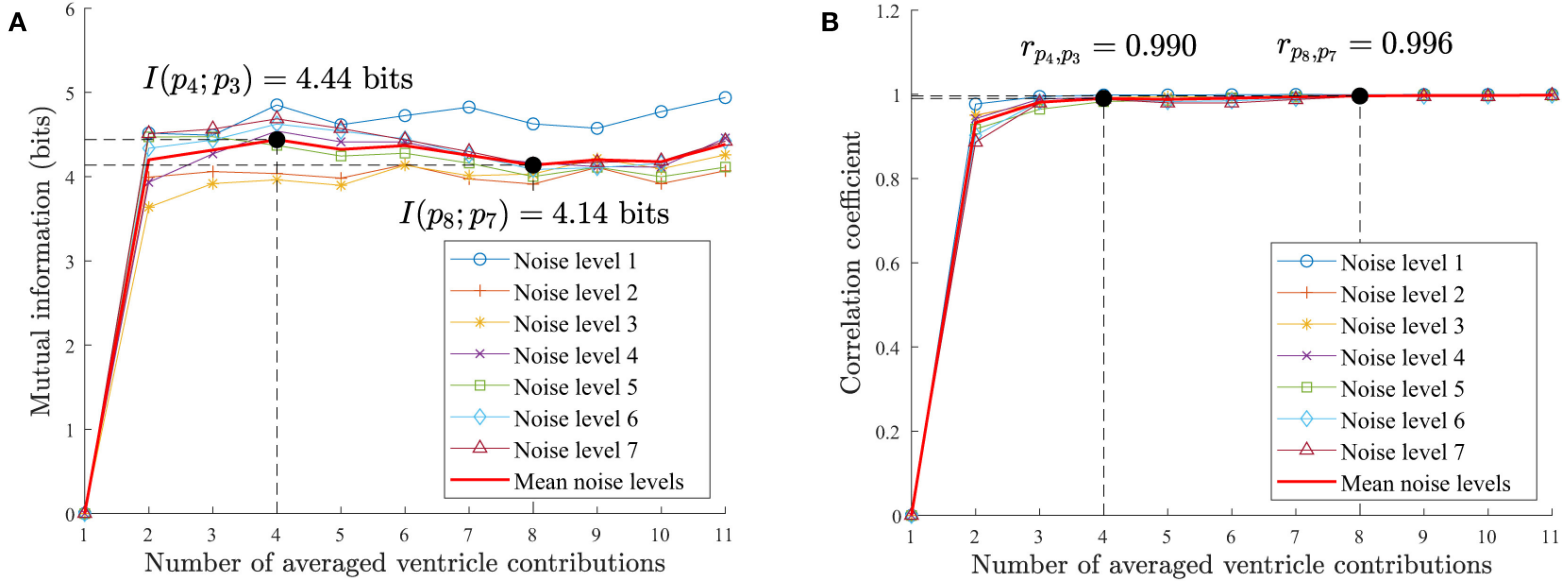
Synthetic AF EGM database pattern analysis. **(A)** Mutual information for an increasing number of ventricular contributions employed to compute the ventricle pattern. **(B)** Correlation coefficient for an increasing number of ventricular contributions. The pattern improves until *V* = 4 contributions in both measurements. For higher values the correlation does not improve significantly. On the other hand, the mutual information degrades for *V* > 4 contributions as it contains repeated information as the increasing correlation value evidences.

**Figure 7:**
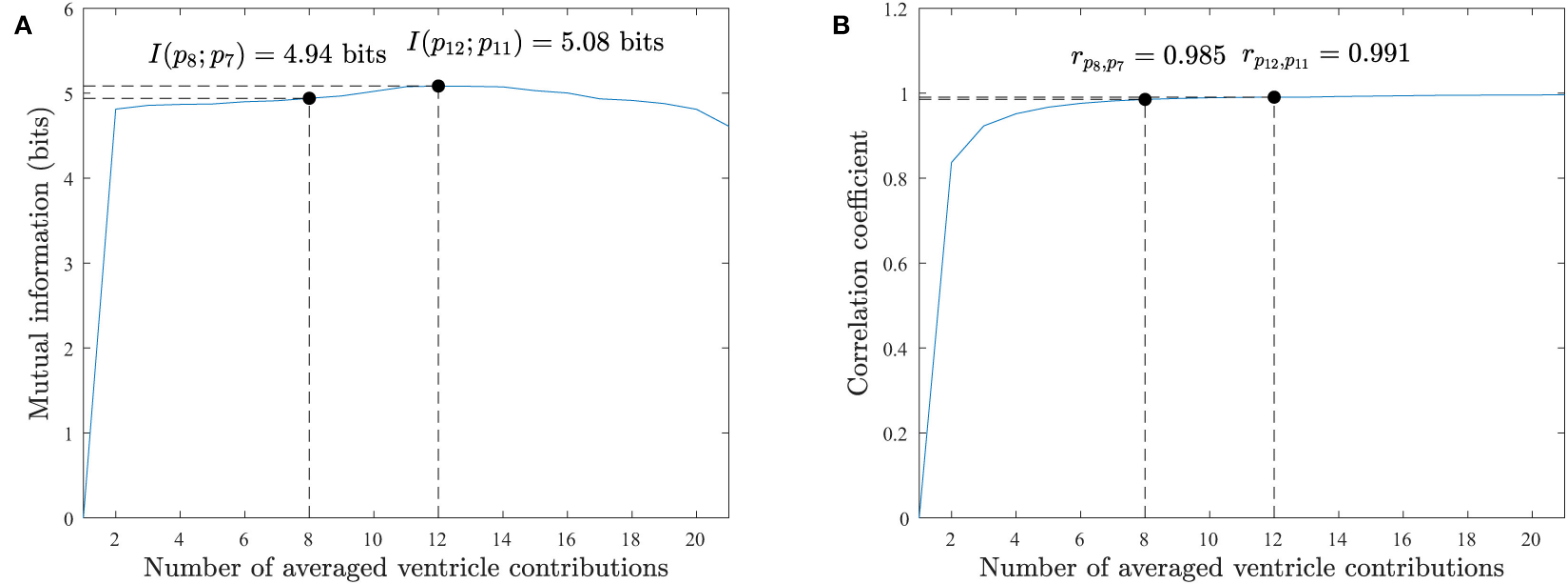
Real AF EGM database pattern analysis. **(A)** Mutual information for an increasing number of ventricular contributions employed to compute the ventricle pattern. **(B)** Correlation coefficient for an increasing number of ventricular contributions. The pattern improves in both measurements as more contributions are used, but for *V* > 12 contributions the correlation does not improve significantly and the mutual information degrades. Both measurements agree with the mutual information and correlation results on the synthetic database in Figure 6.

Following this methodology, the pattern becomes more stable when the mutual information converges to a maximum value. In the synthetic data the mean mutual information for all the noise levels is maximum at *V* = 4 contributions with *I*(*p*_4_; *p*_3_) = 4.439 bits in Figure 6A. We see how the mutual information decreases as more contributions are used to obtain the averaged pattern. In our real AF data in Figure 7A, we achieved the maximum at *V* = 12 contributions with *I*(*p*_12_; *p*_11_) = 5.086 bits. For higher number of contributions the pattern does not longer improve and mutual information decays.

Similarly to mutual information, the correlation of patterns for an increasing number of averaged ventricular contributions will increase as the patterns become more similar to each other. The maximum correlation value *r*_*p*_*i*_, *p*_*i*+1__ = 1 will be reached when the patterns are identical.

The synthetic database achieves the maximum correlation point for *V* = 8 contributions, *r*_*p*_8_, *p*_7__ = 0.996 in Figure 6B. For *V* > 4 contributions the correlation value slows down as it converges to 1. The real AF EGMs database achieves the maximum correlation value for *V* = 12 contributions, *r*_*p*_12_, *p*_11__ = 0.991 in Figure 7B. Once again, for fewer number of contributions, i.e., *V* > 8, the correlation value does not improve significantly as it converges to 1.

### 2.6. Time Domain Approach to Compare Cancellation Methods

The real-time RVC method is compared to the offline ABS algorithm (Shkurovich et al., 1998). We evaluated the performance based on the detection of unipolar EGM LATs after ventricular cancellation on the annotated synthetic database. Both methods share concepts like the averaged pattern calculation, they are similar to implement, and involve analogous signal processing operations, e.g., signal alignment or pattern subtraction. They handle as input signals the same unipolar EGMs and a reference ECG. The F1score for LAT detection was used as the metric for comparison.

The F1score is a standard statistical performance measure obtained as the harmonic mean of sensitivity and precision, also know as true positive ratio (TPR) and positive predictive value (PPV), respectively:

(2) $$\begin{array}{l} TPR=\frac{TP}{P}=\frac{TP}{TP+FN}, \end{array}$$

(3) $$\begin{array}{l} PPV=\frac{TP}{TP+FP}, \end{array}$$

where true positive (TP) is the number of correctly detected LATs, false negative (FN) is the failure when detecting existing LATs, false positive (FP) is the detection of an non-existent LAT, and *P* the number of annotated LAT events. Hence the F1score is calculated as:

(4) $$\begin{array}{l} \text{F1score}=\frac{TPR\times PPV}{TPR+PPV}=\frac{2TP}{2TP+FP+FN}. \end{array}$$

Thousands of simulations were performed for the different parameters involved in the implementation of the RVC algorithm. The analyzed variables were the LAT detection parameters *M*, σ_*abs*_, and τ which correspond to the slope approximation filtering parameter, the minimum detection threshold and the exponential adaptive threshold, respectively as in Ríos-Muñoz et al. (2018), and as an additional parameter for the *RVC* method the number of ventricular contributions *V*. Table 1 includes all the analyzed parameter values, for a total of 11,310 parameter combinations. The performance F1score was calculated on the synthetic LAT database prior to employ the best parameters with real AF EGMs. The first ventricular far field component of the synthetic signals was canceled using a pre-computed pattern using eight different random contributions from the dataset. This approach mimics the behavior of the real-time implementation of the RVC method, which always has an available pattern as the catheter is moving inside the atrial chambers.

**Table 1:** Employed parameters and the values for the 11,310 different combination of settings simulated in the validation of the RVC method on the synthetic signal database, and LAT detection F1score for the different noise level values using the annotated synthetic EGM database for the ABS and RVC methods.

**Parameter**		**Values [Start value:Increment:End value]**					**Number of values**	
*M*		[1:1:10]					10	
σ_*abs*_		[0:0.0001:0.001, 0.002:0.001:0.01, 0.02:0.01:0.2]					39	
τ		[0.0001:0.0001:0.001, 0.002:0.001:0.02]					29	
**Total combinations**							11,310	
**Method**	**Noise levels**							**Mean value**
	**Level 1**	**Level 2**	**Level 3**	**Level 4**	**Level 5**	**Level 6**	**Level 7**	
**LAT DETECTION ON ALL SYNTHETIC EGMs**								
ABS	**0.9533**	0.7454	0.7934	0.7343	0.6275	0.5916	0.5400	0.7125
RVC (V=1)	0.7077	0.5536	0.5894	0.5425	0.4688	0.4211	0.3912	0.5249
RVC (V=2)	0.8566	0.7090	0.7282	0.6965	0.6332	0.5431	0.5085	0.6679
RVC (V=3)	0.9421	0.7363	0.7667	0.7178	0.6343	0.5641	0.5278	0.6984
RVC (V=4)	0.9300	0.7661	0.7859	0.7324	0.6620	0.5787	0.5349	0.7129
RVC (V=5)	0.9344	0.7815	0.7940	0.7335	**0.6743**	0.5833	0.5389	0.7200
RVC (V=6)	0.9366	0.7765	0.7968	**0.7360**	0.6670	0.5928	0.5511	0.7224
RVC (V=7)	0.9418	**0.7837**	0.7961	0.7540	0.6659	0.5985	0.5586	0.7284
RVC (V=8)	0.9402	0.7819	0.7976	0.7526	0.6705	**0.6089**	0.5625	**0.7307**
RVC (V=9)	0.9434	0.7772	0.7950	0.7557	0.6709	0.6075	0.5636	0.7304
RVC (V=10)	0.9444	0.7815	**0.7980**	0.7540	0.6670	0.6073	0.5626	0.7307
RVC (V=11)	0.9417	0.7782	0.7949	0.7526	0.6691	0.6062	**0.5643**	0.7296
**LAT DETECTION RESTRICTED TO EGM SEGMENTS AFFECTED BY FAR-FIELD**								
ABS	**0.9254**	0.7121	0.7399	0.7321	0.6119	**0.6324**	0.5811	0.7050
RVC (V=1)	0.2198	0.1854	0.1870	0.1492	0.1603	0.1426	0.1368	0.1687
RVC (V=2)	0.7346	0.5922	0.6209	0.5994	0.5503	0.4874	0.4502	0.5764
RVC (V=3)	0.9077	0.6667	0.7138	0.6574	0.5793	0.5438	0.5182	0.6553
RVC (V=4)	0.8925	0.7088	0.7314	0.6879	0.6321	0.5716	0.5288	0.6790
RVC (V=5)	0.9036	0.7276	0.7439	0.7004	**0.6557**	0.5701	0.5421	0.6919
RVC (V=6)	0.9069	0.7238	**0.7511**	0.7202	0.6540	0.6017	0.5629	0.7029
RVC (V=7)	0.9169	0.7313	0.7458	0.7306	0.6331	0.6151	**0.5880**	0.7087
RVC (V=8)	0.9135	0.7306	0.7496	0.7377	0.6435	0.6207	0.5811	0.7110
RVC (V=9)	0.9210	0.7255	0.7440	**0.7467**	0.6466	0.6239	0.5837	0.7131
RVC (V=10)	0.9230	**0.7319**	0.7437	0.7416	0.6487	0.6243	0.5859	**0.7142**
RVC (V=11)	0.9177	0.7293	0.7450	0.7414	0.6536	0.6259	0.5857	0.7141

*Performance is measured for all the LATs in the EGMs and also restricting the LAT detection to the segments affected by ventricular far-field. Bold values represent the best F1score performance for each noise level*.

To compare the RVC method in real AF signals, the number of ventricular contributions was fixed to *V* = 8 regarding the performance results for the synthetic signal database in Table 1. Although a value of *V* = 2 contributions shows no relevant differences in mutual information in Figure 7, its pattern does exhibit higher voltage variations than patterns with greater *V* values, as Figure 5 shows. What is more, using *V* = 8 contributions instead of 12 the necessary time that the catheter needs to be deployed and stabilized before acquiring the signal is reduced by a 33% factor. Hence the clinical procedure duration is minimized. Correlation values on the synthetic and real EGM databases present saturated values of correlation around 0.98 − 0.99, meaning that patterns are very similar for an increasing number of averaged ventricular contributions.

The cancellation was performed online while the signals were being acquired directly from the patients using the acquisition system detailed in the Methods section. For the ABS method, the signal processing was performed offline. We used all the QRST contributions in the 10 s signals to calculate the pattern and we applied the cancellation.

**Figure 8:**
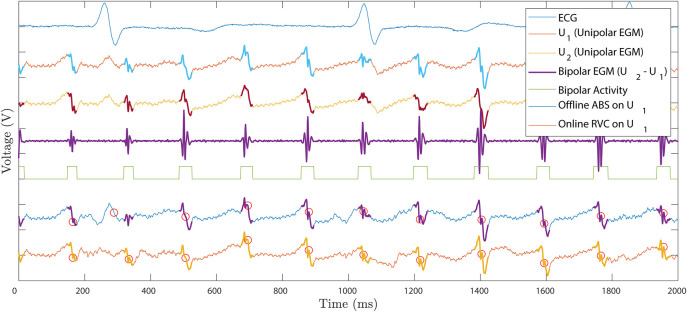
Ventricle cancellation for one unipolar EGM registered at the posterior wall of the left atrium. Top to bottom: ECG reference signal identifying QRST fiducial points. Raw unipolar EGM *U*_1_ where ventricle cancellation is applied. Unipolar EGM *U*_2_ used to compute the bipolar EGM. Differential bipolar signal obtained as *U*_2_ − *U*_1_. Bipolar activity mask, active when there is signal in the bipolar EGM and zero otherwise. Offline ABS method applied to *U*_1_ with LATs circled in red. Online RVC method applied to *U*_1_ with LATs circled in red. Bipolar activity is highlighted in the unipolar EGMs to illustrate the scope of the atrial activation.

Figure 8 contains an example of the ventricular cancellation using both methods. In the top ECG signal we can identify two ventricle activations. The ABS and the RVC outputs applied to the *U*_1_ signal correspond to the signals at the bottom of Figure 8. By visual inspection both methods manage to cancel the ventricular contribution. However, there is no straightforward error measurement to compare their performance. We propose to use the local activity information contained in the bipolar EGM to quantify true atrial activations. Following this reasoning, we can quantify the number of LATs after the ventricular cancellation related to the bipolar activity. Whenever there is an activation in the bipolar EGM, atrial activity exists in the unipolar recording.

Unfortunately, there is no consensus when defining the LAT in a bipolar EGM. As opposed to the unipolar case, where the LAT corresponds to the maximum negative slope of the signal, i.e., $-\frac{dV}{dt}$, the bipolar case remains unclear. Bipolar LATs can be identified as the mean value between bipolar onset-offset (El Haddad et al., 2013), as the maximum negative or positive slope of the signal (De Bakker et al., 1995), or even identified by a predefined annotated signal database (Treo et al., 2013). In the following, we define the presence of bipolar activity when the bipolar EGM contains energy over a predefined threshold. We take into consideration the temporal window of the bipolar activations and generate a binary bipolar mask *b*[*n*], i.e., *b*[*n*] = 1 if there is activity and *b*[*n*] = 0 otherwise, see the squared pulse train in Figure 8. To obtain the binary bipolar mask the EGM is filtered similarly to El Haddad et al. (2013).

After the mask, the LATs in the ventricular-free unipolar signals are detected for both ABS and RVC methods. A LAT detection algorithm enhances the slope of the voltage signal prior to the detection of its maximum negative slope (Ríos-Muñoz et al., 2018). The best performance RVC method parameter combination was used.

### 2.7. Frequency Domain Approach to Compare Cancellation Methods

We investigated the frequency spectrum impact of the ventricular cancellation methods with respect to the original raw EGMs. We computed the average dominant frequency (DF) of the signals using the settings in Botteron and Smith (1995). We also analyzed the average energy spectrum ratio in the EGM signals for two frequency bands 3–5.5 and 5.5–12 Hz that quantifies the performance of the T-wave subtraction (Salinet et al., 2013). The *P*_*ratio*_ is defined as

(5) $$\begin{array}{l} P_{ratio}=\frac{P_{5.5-12Hz}}{P_{3-5.5Hz}}=\frac{\int_{5.5}^{12}P\left(f\right)df}{\int_{3}^{5.5}P\left(f\right)df}, \end{array}$$

where *P*_*a*−*b*_ is the area under the power spectrum curve for the frequency interval *a* and *b* in Hz, and *P*(*f*) is the frequency spectrum function of the EGM signal being analyzed. The *P*_*ratio*_ aims to measure the quality and global effect of the ventricular cancellation methods. The higher the value of the *P*_*ratio*_, the better the T-wave repolarization subtraction is achieved. Table 2 contains the results.

**Table 2:** Frequency analysis of the ABS and RVC cancellation methods.

**Signal**	**DF Mean (Hz)**	**DF Standard deviation (Hz)**	***P*_*ratio*_ Mean**	***P*_*ratio*_ Standard deviation**
**SYNTHETIC AF SIGNALS DATABASE**				
Raw EGM	4.8645	0.8788	1.6959	0.3040
ABS	5.1784	1.2339	1.7638	0.4011
RVC	5.0619	1.1581	1.7175	0.3470
**REAL AF SIGNALS DATABASE**				
Raw EGM	5.4119	0.9412	1.8851	0.7321
ABS	5.7065	0.8314	2.1914	0.9686
RVC	5.9276	0.9350	2.4644	1.1942

*DF and P_ratio_ analysis results are given in mean and standard deviation for the synthetic and real AF signal databases*.

### 2.8. Phase Domain Approach to Compare Cancellation Methods

To complete the study of the methods, we also investigated the phase domain of the signals. Phase mapping is commonly used to analyze AF signals regarding reconstruction of the atrial wavefronts and rotor singularity points detection (Narayan et al., 2012). We analyzed the synthetic signal database and applied the phase mapping method by Kuklik et al. (2015). The method uses sinusoidal recomposition for unipolar EGMs based on the Hilbert Transform of the signal, where the output signal is the combination of different sinusoidal wavelets with amplitude proportional to the EGM negative slope. We followed a similar approach as in Kuklik et al. (2015) to assess the performance of the methods in the phase domain. We compared the cycles of the annotated intervals in the database and compared them to the time instants at which the phase value of the ABS and RVC signals is inverted, which are known to exhibit a good correlation (Kuklik et al., 2015). We computed the mean absolute error as performance metric for all the noise levels in the synthetic database and included the results in **Table 4**.

## 3. Results

This section is devoted to the performance results of the RVC and ABS methods in synthetic and real AF signals. Firstly, we report the ventricular cancellation performance of the RVC method to the ABS for the synthetic database signals for LAT detection. Secondly, we present the frequency domain analysis results of the methods. Then, phase mapping results for both methods is investigated. Finally, we present the results of applying the RVC on real AF signals in real-time.

### 3.1. Local Activation Time Detection Results

#### 3.1.1. Synthetic AF EGMs

**Figure 9:**
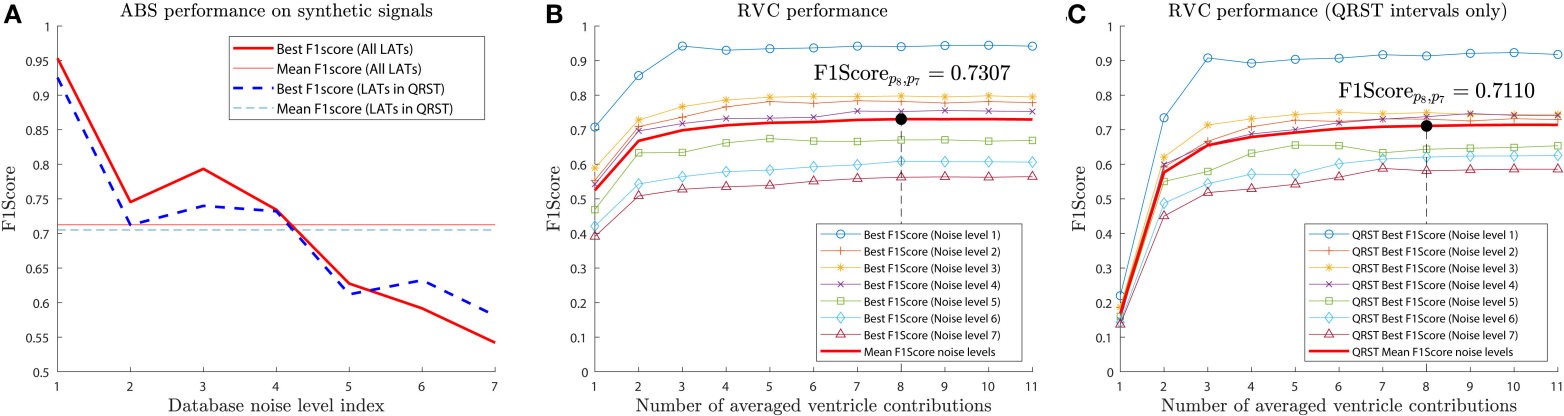
Average F1score performance for the synthetic signal database. Performance is greatly affected by the noise level present in the signals. **(A)** ABS method scored a mean F1score of 0.7125 for all LATs and 0.7050 for the activations affected by the far field. **(B)** RVC method achieved a mean F1score of 0.7307. **(C)** RVC method obtained a mean Fscore value of 0.7110 for LAT detection in the far field affected intervals.

Figure 9A presents the best F1score results for the ABS method for the different noise levels of the synthetic database sets. As expected performance degrades as the noise level increases, exhibiting an average *F*1*Score* = 0.7125. For the RVC method the same parameter combination simulations were employed, including the additional *V* number of ventricular contributions parameter. The total number of simulations for the RVC was 124,410. Figure 9B shows the analysis for the different values of contributions and the different noise levels. The best average performance was obtained for *V* = 8 contributions with *F*1*Score* = 0.7307.

We included the performance of the LAT detection taking into account only the activations within the interval affected by the far field. The ABS method achieved an average *F*1*score* = 0.7050 (Figure 9A), and the RVC method *F*1*score* = 0.7110 (Figure 9C).

Table 1 summarizes the simulation results for both methods. In the absence of noise both methods perform similarly, but globally the RVC method performs better and specially for clinical EGMs that suffer from additive noise. Parameter settings for the RVC method were: *M* = 6, $\sigma_{abs}=1\cdot 10^{-4},\text{ and }\tau =1\cdot 10^{-4}$. The best parameter settings for the ABS were achieved for: *M* = 8, $\sigma_{abs}=0,\text{ and }\tau =3\cdot 10^{-4}$.

#### 3.1.2. Real AF EGMs

The number of detected LATs in the signals was quantified, and the performance of the methods only during the QRST interval was evaluated. Table 3 shows that both methods achieve similar results, with the RVC method detecting more and better LATs in both the whole 10 s EGMs, and additionally for the EGMs segments comprised in the QRST intervals. One of the reasons the ABS provides a lower number of LATs is related to the number of ventricular activations that are used to compute the averaged pattern. In some signals the number of QRST contributions might be too high (up to 21 in our database as shown in Figure 7), and since the AF nature is indeed irregular, the atrial recording can vary in time which can worsen the quality of the pattern.

**Table 3:** RVC and ABS LAT detection performance for the real AF signals database.

**Method**	**Signals**	**Total LATs**	**Inside bipolar mask**
ABS	Complete EGMs	578,404	550,260 (95.13%)
RVC	Complete EGMs	**581,992**	**556,084 (95.54%)**
ABS	Only QRST range	304,353	292,584 (96.13%)
RVC	Only QRST range	**309,344**	**297,651 (96.22%)**

*Bold values represent the method that detected more LATs*.

Some of the undetected LATs in the bipolar mask corresponded to activations associated to multiple deflections in the unipolar EGM that were detected before or after the bipolar binary mask. This is an expected result of unipolar configuration, since these activations might be associated to double potentials of the atrial tissue, activations when the catheters touch each other inside the atrial chamber, fragmented EGMs related to electrical remodeling of the cardiac tissue, or even to additional muscular far-field.

### 3.2. Frequency Analysis Results

For the synthetic database, the mean DF for raw EGMs, the ABS method and the RVC method were 4.4864, 5.1784, and 5.0619 Hz, respectively. For the real AF signals, the mean DF for the raw EGMs, the ABS method and the RVC method were 5.4119, 5.7065, and 5.9276 Hz, respectively. All DF distributions were compared with a paired *t*-test and *p*-values were obtained. Significance was assumed if *p*-value < 0.01. Results in Table 2 show that the DF for both cancellation methods is significantly increased with respect to the raw real AF EGMs (both ABS and RVC *p*-values were smaller than 0.01).

The *P*_*ratio*_ results in Table 2 also provided evidence that the ventricular cancellation methods had an impact in the frequency spectrum. In the synthetic database, the mean *P*_*ratio*_ for the raw EGMs was 1.6959, whereas the ABS method achieved 1.7638 and the RVC 1.7175. For the real AF signals, mean *P*_*ratio*_ for the raw EGMs was 1.8851, whereas the ABS method achieved 2.1914 and the RVC 2.4644. Significance *t* test between the methods distributions and the raw signals confirmed the significance of the results (*p*-value < 0.01.) The *RVC* method achieved the highest mean *P*_*ratio*_.

### 3.3. Phase Mapping Analysis Results

We applied phase mapping to the synthetic database signals to evaluate the impact of the ventricular cancellation methods on the phase domain. We compared the mean cycle length of the signals calculated from the annotated LATs and the mean cycle obtained from the phase inversion times of the phase signal as proposed in Kuklik et al. (2015). The results in Table 4 show evidence of the good correlation between the phase mapping intervals and the time domain for low noise level signals. For noise level 1 the ABS method obtained a mean absolute error of 1.2678 ms and the RVC method scored 0.9189 ms. Error was calculated as the mean of the absolute error of all the independent intervals. The performance worsened for higher noise levels, in which the RVC method obtained lower error values compared to the ABS. Overall phase results showed that the mean error of the ABS was 16.9832 ms and the RVC 15.8243 ms for the complete database.

**Table 4:** RVC and ABS phase mapping study on the cycle length for the synthetic AF signals database.

**Method**	**Noise levels phase cycle mean (ms)**							**Mean value**
	**Level 1**	**Level 2**	**Level 3**	**Level 4**	**Level 5**	**Level 6**	**Level 7**	
True mean cycle	244.6185	241.7837	241.9453	244.9179	241.7709	244.9179	244.9179	243.5532
ABS	244.4202	228.2415	228.0876	229.4916	221.1036	220.1026	222.1704	227.6596
RVC (V=8)	245.2922	225.3699	232.4709	231.4946	223.0215	227.8416	221.8351	229.6180
**Method**	**Noise levels mean phase cycle absolute error (ms)**							**Mean value**
	**Level 1**	**Level 2**	**Level 3**	**Level 4**	**Level 5**	**Level 6**	**Level 7**	
ABS	1.2678	**15.1724**	15.1383	**16.7363**	21.872	25.045	23.651	16.9832
RVC (V=8)	**0.9189**	17.4484	**11.0148**	19.353	**20.0726**	**18.8794**	**23.0828**	**15.8243**

*Cycle lengths are calculated with respect to the phase inversion time instants and they are compared to the true cycle length from the annotated database. Error metrics are expressed as mean absolute values in ms. Bold values represent the method that achieved the lowest mean phase cycle absolute error value for each of the noise levels*.

## 4. Discussion

The most widely used method for ventricle cancellation in unipolar AF signals is ABS. The ABS method is applied offline, which introduces a delay ranging from a few seconds to even minutes depending on the implementation and the length of the acquired signals. To reduce this latency we need an online method instead of one that is applied to batches of signals or lengthy acquisitions. What is more, the method must be predictive to not depend on complete QRST ECG complex detection to be applied.

In consequence, the proposed RVC method introduces an important innovation in the cardiac electrophysiology field, since it estimates the ventricular pattern affecting the unipolar atrial signal based on previous stored contributions in real-time. This feature avoids the complete QRST interval detection requirement of other methods and it enables online signal processing, the main contribution of this paper. This advantage allows other algorithms to receive signals at reduced delay boosting their response time, e.g., real-time rotor detection (Daoud et al., 2017; Ríos-Muñoz et al., 2018), dominant frequency analysis (Botteron and Smith, 1995), or causality EGM relationships (Luengo et al., 2019). To evaluate the RVC method, the quality of the patterns was analyzed with respect to the number of averaged contributions with a novel approach based on mutual information and correlation measurements (see Figures 6, 7). Although it may seem sensible to use as many contributions as possible, obtaining a pattern with a higher number of contributions is not advisable, as our results confirmed. Firstly because of safety reasons, since the catheter would be required to be placed for a longer period at the same position, extending the overall duration of the clinical procedure. What is more, certain areas of the atria are more difficult to map, and providing a stable position with the catheter becomes a challenge for lengthy acquisitions. Secondly, due to the computational time cost for processing more contributions, which also increases memory buffer allocation in the system. We consider that 8–12 contributions provide a good pattern for the real-time ventricle cancellation, while preserving the quality of the pattern. The results on the synthetic and real AF EGM support this choice.

In this sense, we provided a safe way to validate the RVC method using a LAT annotated unipolar EGM database. We quantified the number of true detected LATs based on the activity of bipolar EGMs. We concluded that our method was able to detect more and better the LATs compared to the ABS approach. We can relate this behavior to the number of ventricular contributions employed to calculate the cancellation patterns. The more contributions that are used the worse the pattern becomes, which is the case of cancellation methods that use higher number of contributions, as the mutual information and correlation measurements confirmed in Figure 7.

As for the real AF EGM database, we are aware of potential limitations when using bipolar signals which are very sensitive to the electrical wavefront directionality with respect to the differential electrodes calculating the bipolar measurement. As an example, if two electrodes are activated at the same time the resulting bipolar signal will contain no electrical activity information since the signals cancel each other. For this reason we cannot conclude that the activations that do not match the binary bipolar mask correspond to false atrial activity or spurious deflections derived from the ventricle cancellation method. We are also aware that the binary bipolar mask proposed works with regular bipolar EGMs, but fragmentation might affect this methodology. To that end we resorted to validate the method using a pre-existing annotated database of unipolar LATs, which granted us the best parameter values to tune our RVC method for different noisy signals. The ABS and RVC methods behave similarly, but the LAT performance and its real-time implementation, support the choice of RVC over ABS.

The frequency domain analysis of the method also provides evidence that the method achieves to cancel the ventricular contributions as the value of *P*_*ratio*_ is larger than the original signals. In terms of frequency performance, both methods managed to provide similar results, so the preference of RVC over ABS is justified because of its online and low delay implementation.

The phase domain provided smaller error values for low signal-to-noise in Table 4. As expected, as noise level increased the error metric became worse, due to the multiple deflections introduced by the additive noise in the signals. Phase mapping is a powerful and useful technique to analyze AF EGMs, however in the presence of noise or abundant fragmentation, its performance degrades and it is not advisable to use it due to the low correspondence between the phase transitions and temporal activations. In terms of phase information both ABS and RVC methods behave similarly, so similarly to the frequency results, the real-time feature of the RVC is the main reason for preferring it over ABS.

**Figure 10:**
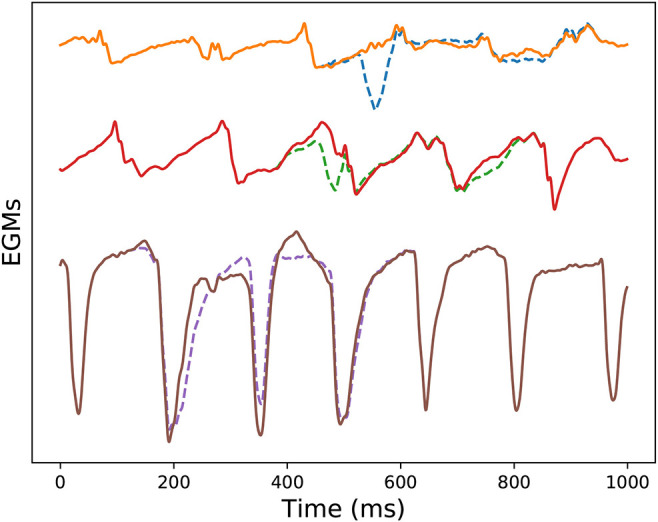
RVC on three different acquisitions, buffer size was *Q* = 6 (600 ms). From top to bottom: electrode at the left superior pulmonary vein, posterior wall of right atrium, and left atrial appendage. Dashed signals represent the unipolar EGMs containing ventricle contribution, and continuous lines represent the RVC outcome.

Finally, we include the output of the real-time ventricle cancellation algorithm applied to three signals exhibiting different morphology in Figure 10. The EGM database consists of signals recorded in all the areas of the left atrium, e.g., pulmonary veins, left atrial appendage, or posterior wall. In the example the dashed signals represent the signals before the RVC algorithm, and the continuous ones free of ventricular contribution. For low voltage signals the effect of the ventricle on the atrial activations is larger if compared to an EGM near a high voltage signal like the areas surrounding the atrial appendages. For these high energy atrial signals we see how the real-time ventricle cancellation has little effect. But it is for the rest of low atrial energy signals when the ventricle cancellation becomes more useful. In Figure 10 the first two signals exhibit a higher ventricular contribution, and the real-time cancellation achieves to recover the hidden atrial information. Additional examples of different atrial sites are included in Supplementary Figures 3, 4.

## 5. Conclusions

We have presented a real-time signal processing method that predicts and removes the far-field activity present in unipolar EGMs recorded during AF. As opposed to other offline methods, the RVC method reduces the delay to process the signals, which allows real-time cancellation. The online cancellation method has been tested on synthetic and real AF EGMs. We have compared its performance to other existing offline method for LAT detection after ventricular cancellation, and we also investigated the cancellation impact on the frequency and phase domains. The online method achieved better results, was more robust, and its implementation in the clinical practice provided real-time results for simultaneous multi-electrode AF signals. In the clinical environment other online investigations, e.g., rotational activity assessment, dominant frequency or local activation time mapping, might benefit from the real-time potential of the proposed cancellation method.

## Data Availability Statement

The synthetic datasets for this study are available upon request to the corresponding author in the article by Valinoti et al. (2018). The code for the RVC method presented in this paper is also available upon request to the corresponding author.

## Ethics Statement

All patients gave full informed consent and the study was approved by the local ethical committee of the Hospital General Universitario Gregorio Marañón, Madrid, Spain.

## Author Contributions

ÁA, FF-A, and AA-R acted as supervisors of the research and contributed in methodological aspects. GR-M and ÁA acquired the signals from patients. GR-M was responsible for performing the data analysis and developing the algorithms and methods. All authors contributed equally to the conception and design of the study, contributed to the manuscript revision, read, and approved the submitted version.

## Conflict of Interest

The authors declare that the research was conducted in the absence of any commercial or financial relationships that could be construed as a potential conflict of interest.

## Supplementary Material

Supplementary Figure 1 Real-time ECG fiducial points detection for *V* = 8 ventricular contributions in an AF patient. Example of a buffered ECG lead in which real-time R-peak detection is applied regarding the maximum peak voltage and R-peak threshold, red dots and horizontal line, respectively. Q-peak and *T*_*end*_ detection depend on the R-peak detection and are also displayed in the figure, black crosses and magenta diamonds, respectively.

Supplementary Figure 2 RVC execution times for different values of electrodes calculated simultaneously. The simulations were run 500 times for 100 ms segments for the different number of electrodes. Each of the realizations are represented and the mean execution times for all the electrode values is plotted as a continuous line.

Supplementary Figure 3 Ventricle cancellation using ABS and RVC methods for one unipolar EGM registered at the left atrial appendage. The same figure legend applies as in Figure 8. At this site the ventricular component is weaker because of the high voltage of the left atrial appendage.

Supplementary Figure 4 Ventricle cancellation using ABS and RVC methods for one unipolar EGM registered at the left inferior pulmonary vein. The same figure legend applies as in Figure 8. At this site the ventricular component is stronger.
