# Supplementary figures and images for: Real-Time Ventricular Cancellation in Unipolar Atrial Fibrillation Electrograms
Source: Front Bioeng Biotechnol. 2020 Jul 30;8:789. doi: 10.3389/fbioe.2020.00789 (PMC7406791; doi:10.3389/fbioe.2020.00789)

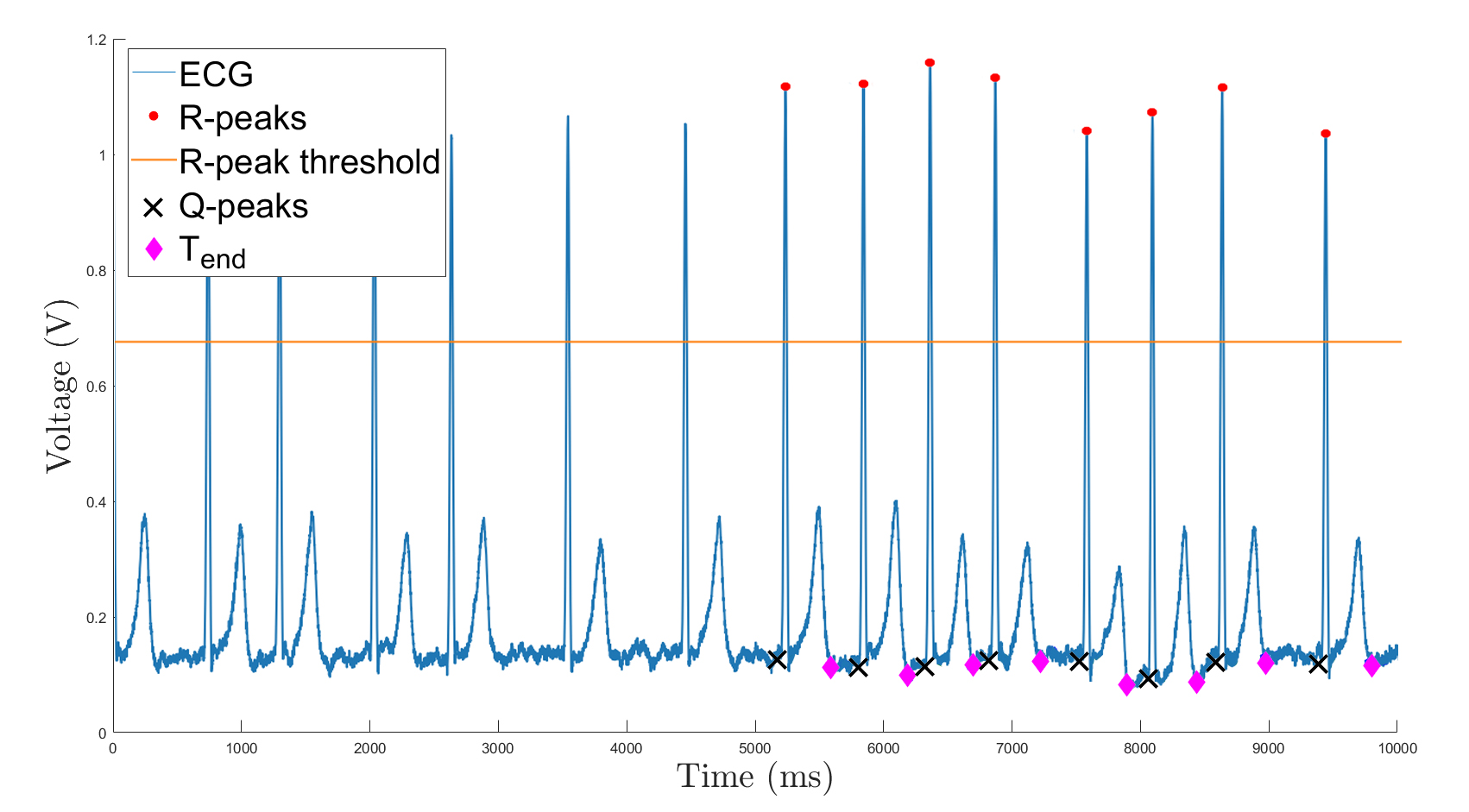

Supplement: Supplementary Figure 1 — Real-time ECG fiducial points detection for V = 8 ventricular contributions in an AF patient. Example of a buffered ECG lead in which real-time R-peak detection is applied regarding the maximum peak voltage and R-peak threshold, red dots and horizontal line, respectively. Q-peak and Tend detection depend on the R-peak detection and are also displayed in the figure, black crosses and magenta diamonds, respectively. [file Image_1.JPEG]

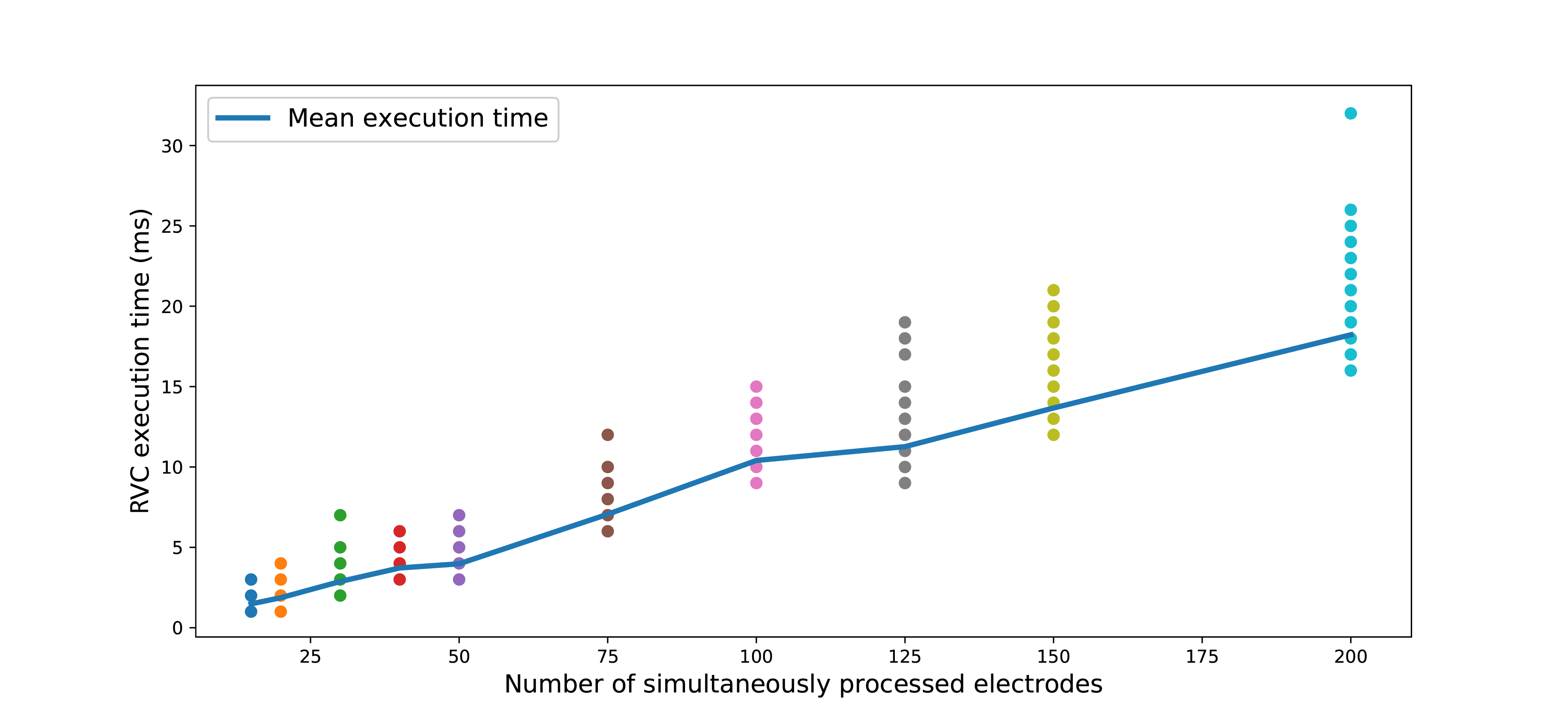

Supplement: Supplementary Figure 2 — RVC execution times for different values of electrodes calculated simultaneously. The simulations were run 500 times for 100 ms segments for the different number of electrodes. Each of the realizations are represented and the mean execution times for all the electrode values is plotted as a continuous line. [file Image_2.JPEG]

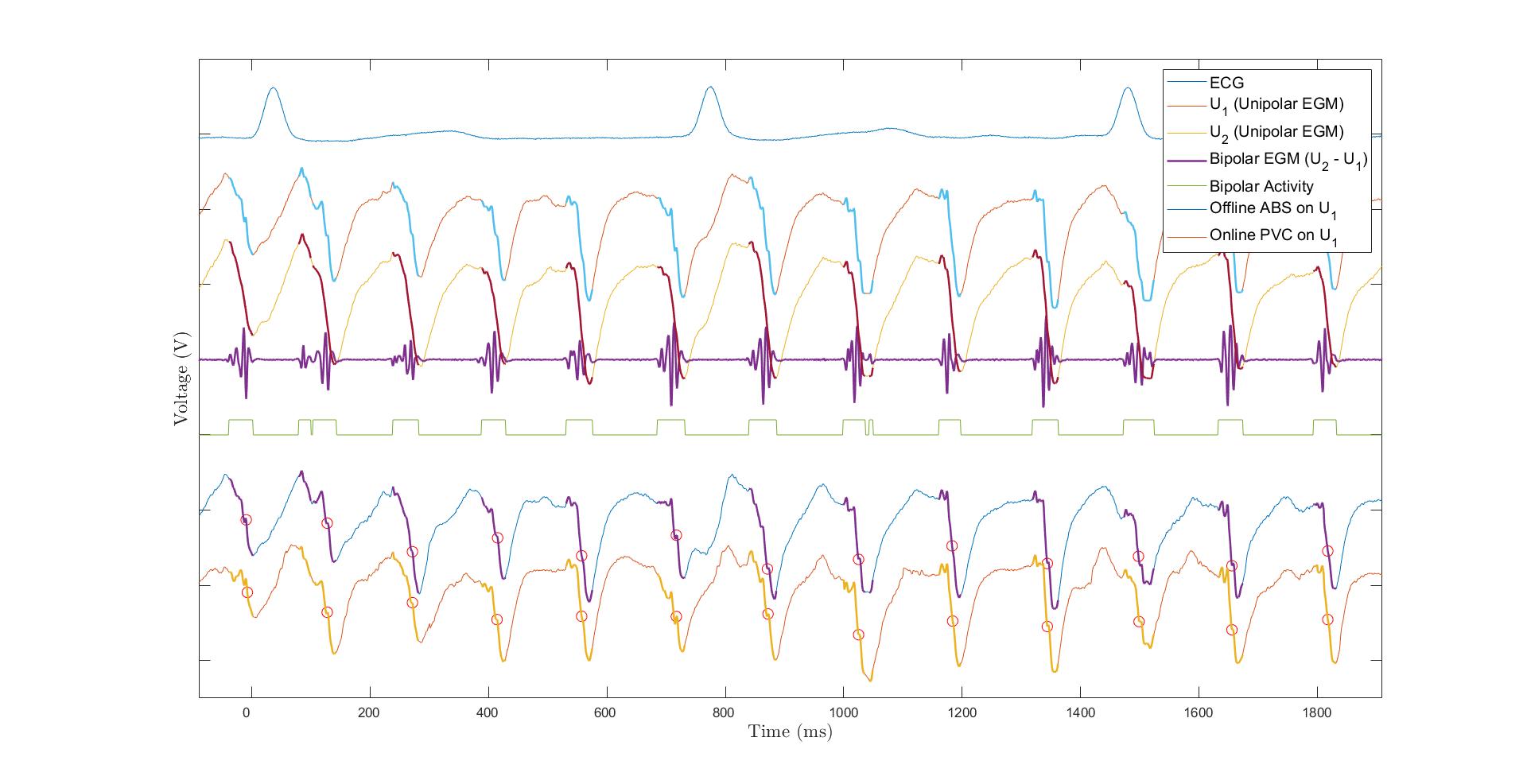

Supplement: Supplementary Figure 3 — Ventricle cancellation using ABS and RVC methods for one unipolar EGM registered at the left atrial appendage. The same figure legend applies as in Figure 8. At this site the ventricular component is weaker because of the high voltage of the left atrial appendage. [file Image_3.JPEG]

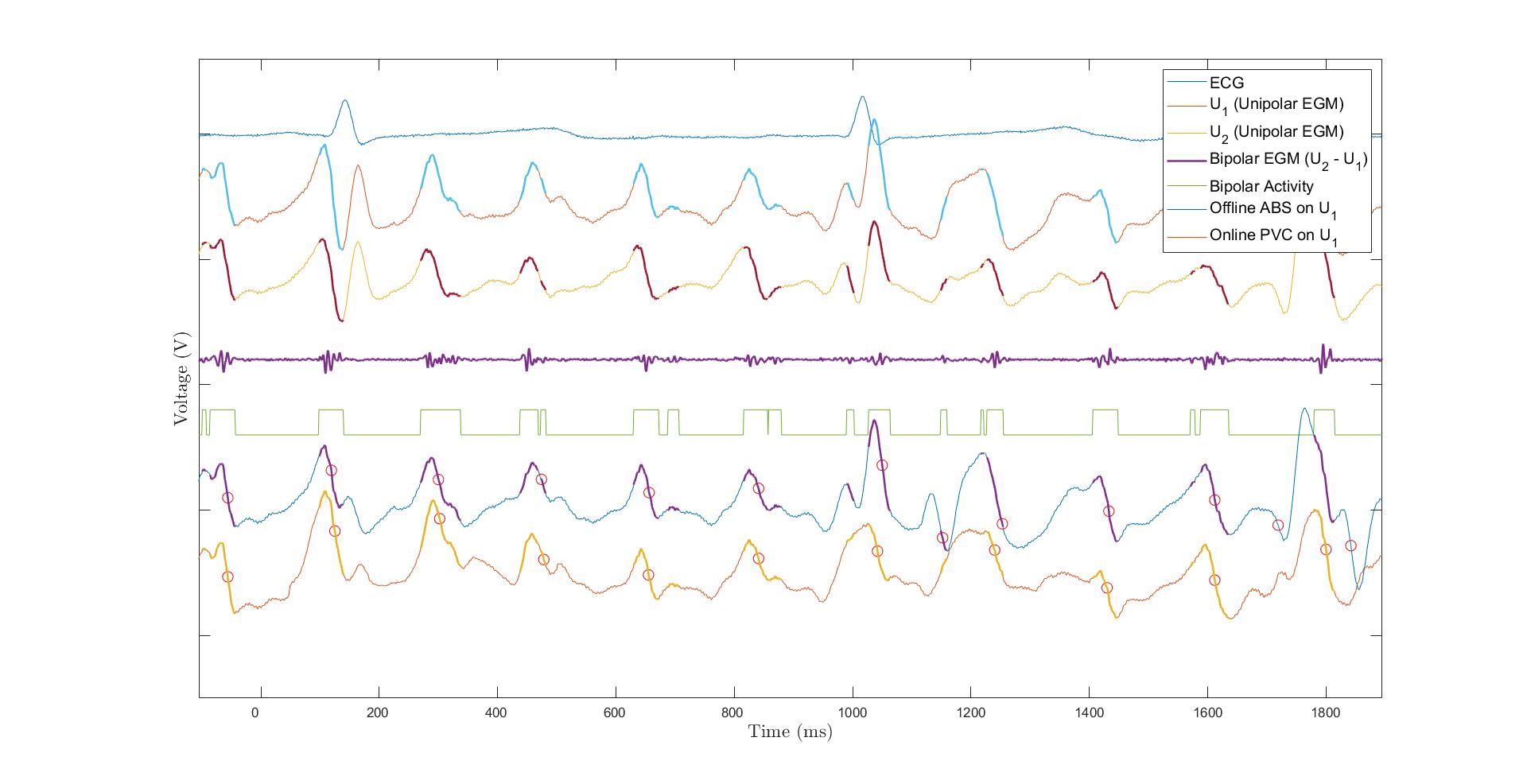

Supplement: Supplementary Figure 4 — Ventricle cancellation using ABS and RVC methods for one unipolar EGM registered at the left inferior pulmonary vein. The same figure legend applies as in Figure 8. At this site the ventricular component is stronger. [file Image_4.JPEG]
